# Diagnosing Balamuthia mandrillaris encephalitis via next-generation sequencing in a 13-year-old girl

**DOI:** 10.1080/22221751.2020.1775130

**Published:** 2020-06-18

**Authors:** Xia Wu, Gangfeng Yan, Shuzhen Han, Yingzi Ye, Xunjia Cheng, Hairong Gong, Hui Yu

**Affiliations:** a Department of Infectious Diseases, Children’s Hospital of Fudan University, Shanghai, People’s Republic of China; b Department of Pediatric Emergency Medicine and Critical Care Medicine, Children’s Hospital of Fudan University, Shanghai, People’s Republic of China; c Department of Medical Microbiology and Parasitology, School of Basic Medical Sciences, Fudan University, Shanghai, People’s Republic of China

**Keywords:** *Balamuthia mandrillaris*, Balamuthia amoebic encephalitis, granulomatous amebic encephalitis, next-generation sequencing, children

## Abstract

Balamuthia amoebic encephalitis has a subacute-to-chronic course and is almost invariably fatal owing to delayed diagnosis and a lack of effective therapy. Here, we report a 13-year-old girl with cutaneous lesions and multifocal granulomatous encephalitis. The patient underwent a series of tests and was suspected as having tuberculosis. She was treated with various empiric therapies without improvement. She was finally correctly diagnosed via next-generation sequencing of the cerebrospinal fluid. The patient deteriorated rapidly and died 2 months after being diagnosed with Balamuthia mandrillaris encephalitis. This study highlights the important clinical significance of next-generation sequencing, which provides better diagnostic testing for unexplained paediatric encephalitis, especially that caused by rare or emerging pathogens.

## Introduction

*Balamuthia mandrillaris*, a free-living amoeba found in soil and water, causes encephalitis in humans, sheep, dogs, horses, and nonhuman primates. *B. mandrillaris* is likely transmitted via inhalation or inoculation through broken skin and spreads to the brain and other organs through haematogenesis. In 1990, *B. mandrillaris* became recognized as a pathogen that causes granulomatous amoebic encephalitis (GAE) in humans. To date, more than 200 cases of Balamuthia amoebic encephalitis (BAE) have been identified worldwide. The disease is usually fatal and is difficult to successfully treat. Here, for the first time in China, we treated a 13-year-old girl with cutaneous lesions and multifocal granulomatous encephalitis. The patient deteriorated rapidly and died 2 months after being diagnosed with *B. mandrillaris* encephalitis via next-generation sequencing (NGS).

## Results

### Case report

**Figure 1.**
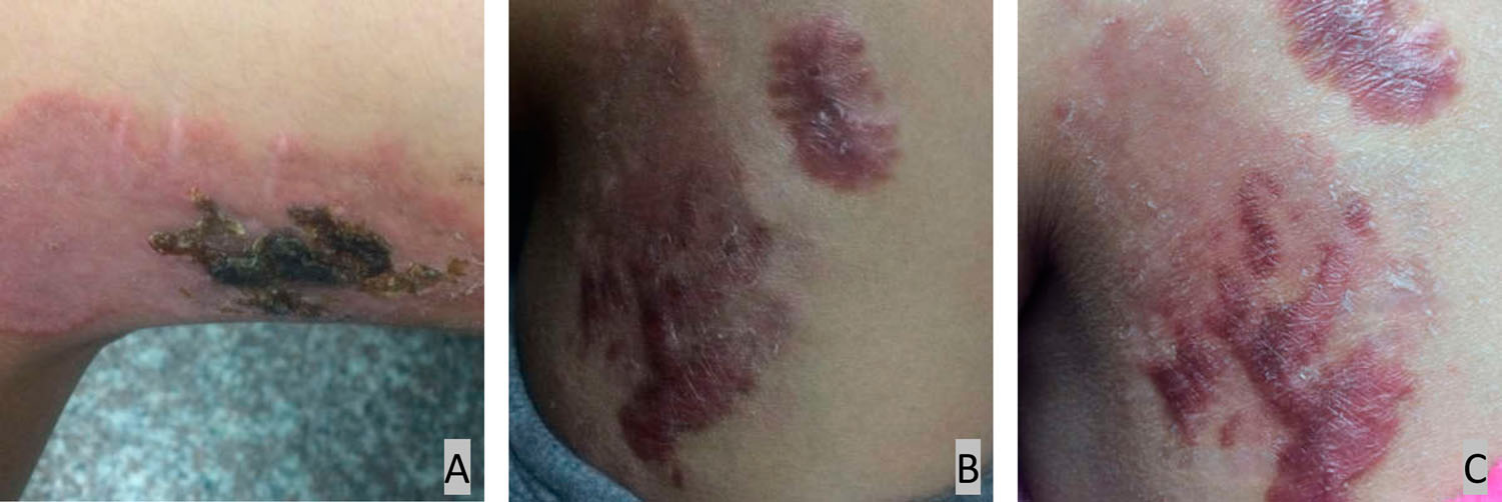
A, Cutaneous lesion on the left observed in September 2018 showing an erythematous plaque; B, Cutaneous lesion on the left observed in March 2019; C, Cutaneous lesion on the left observed in April 2019 showing a crusty surface and scarring.

A previously healthy 13-year-old girl presented in March 2019 with a two-year history of cutaneous lesions on her left thigh. Two years prior, she noticed several asymptomatic papules on her left thigh. The skin lesions slowly increased in size to form an erythematous plaque. Two years later, the lesion on her left thigh became enlarged and evolved into violaceus plaques (Figure 1). A topical steroid cream was administered for 4 weeks without resolution of the skin lesion. A tuberculin skin test was negative. A skin biopsy was performed at another institution in September 2018. Histopathologic examination revealed a granulomatous reaction rich in lymphocytes and was suspected to be tuberculosis. The patient began empirical therapy with rifampin and clarithromycin for 4 months, and the lesions improved.

**Figure 2.**
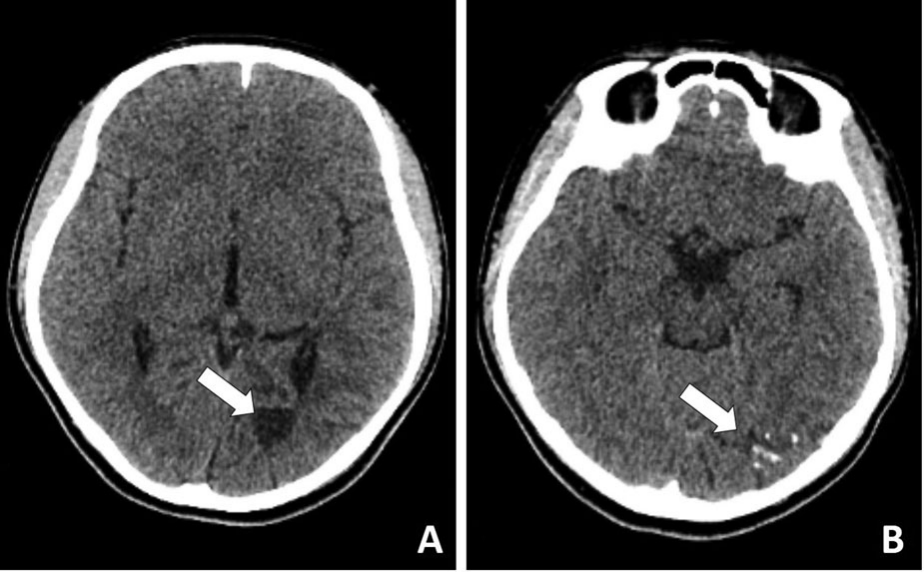
Computed tomography scan of the brain revealed an area of hypodensity in the region of the left lateral ventricle and left occipital lobe.

One month after discontinuing the medication (Feb. 2019), she was taken to a hospital because of dizziness, vomiting, and blurry vision. A computed tomography scan of the brain revealed an area of hypodensity in the region of the left lateral ventricle and left occipital lobe (Figure 2). Three weeks later, the girl developed abnormal eye movements and diplopia. The patient was treated with antimicrobials, including isoniazid, rifampin, ceftazidime, and amikacin. The patient clinically deteriorated with worsening vomiting and headaches.

In March 2019, she was admitted to Children’s Hospital of Fudan University with symptoms of fever, vomiting, headaches, and a right esotropia. Cerebrospinal fluid (CSF) examination showed 100 × 10^6^ leukocytes/L (20% neutrophils, 80% lymphocytes), glucose of 1.6 mmol/L, and protein of 1467 mg/L. CSF cultures showed no bacteria or fungi. Infectious meningitis and encephalitis were suspected, and intravenous meropenem (4.5 g daily) and linezolid (1200 mg daily) were given without improvement.

**Figure 3.**
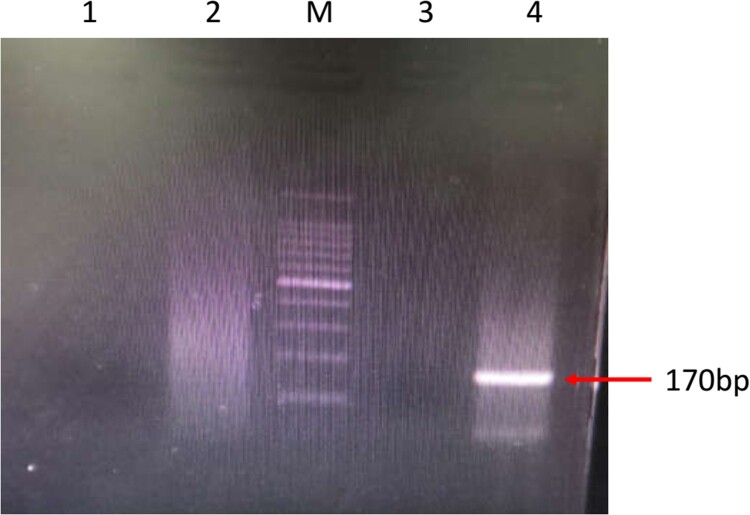
PCR amplification of *Acanthamoeba* and *Balamuthia* DNA using the specific primers, BalaF1451 and BalaR1621. M: 100-bp DNA ladder; Lane 1: negative control detecting *Acanthamoeba*; Lane 2: patient’s sample detecting *Acanthamoeba*; Lane 3: negative control detecting *Balamuthia*; Lane 4: patient’s sample detecting *Balamuthia*

**Figure 4.**
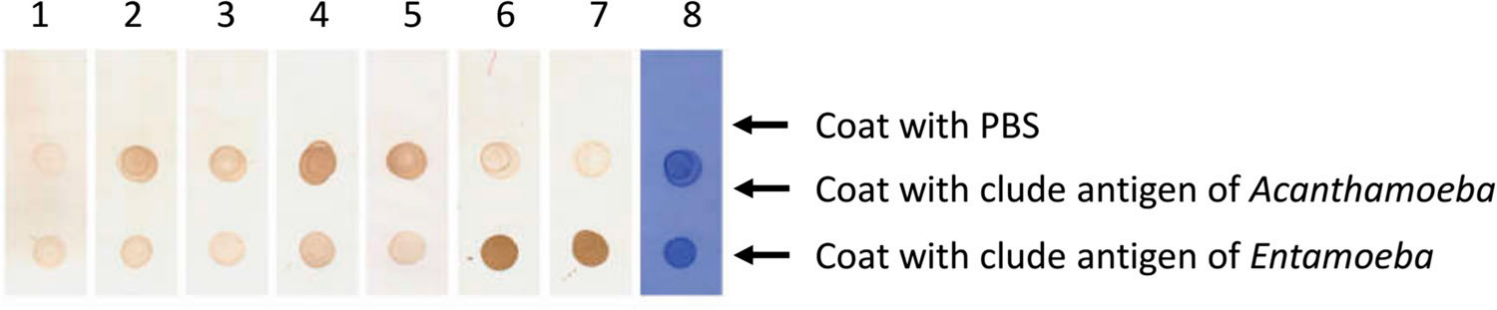
Immunostaining of serum and CSF samples. Lane 1: negative serum (1:50 dilution); Lane 2: patient serum (19-4-1; 1:50 dilution); Lane 3: patient serum (19-4-1; 1:100 dilution); Lane 4: patient CSF (19-4-1; 1:2 dilution); Lane 5: patient CSF (19-4-1; 1:50 dilution); Lane 6: Entamoeba Ag-positive serum (1:50 dilution); Lane 7: Entamoeba Ag-positive serum (1:100 dilution); Lane 8: CBB staining.

Her CSF sample was sent to the Beijing Genomics Institute (BGI)-Wuhan for pathogen detection via NGS. Three days later, NGS of the CSF confirmed the presence of *B. mandrillaris* genomic sequences. One hundred sixty-five sequence copy reads corresponded to *B. mandrillaris*. No bacteria, fungi, viruses, Mycobacterium tuberculosis, or mycoplasma were detected via NGS of the CSF. *B. mandrillaris* was confirmed by polymerase chain reaction (PCR) of a skin sample (obtained from the skin biopsy in Sep. 2018) using the specific primers, BalaF1451 (TAACCTGCTAAATAGTCATGCCAAT) and BalaR1621 (CAAACTTCCCTCGGCTAATCA) (Figure 3). Immunostaining of serum and CSF samples obtained from the patient in Apr. 2019 yielded an Acanthamoeba antibody titre of 1:50–1:100 (Figure 4).

**Figure 5.**
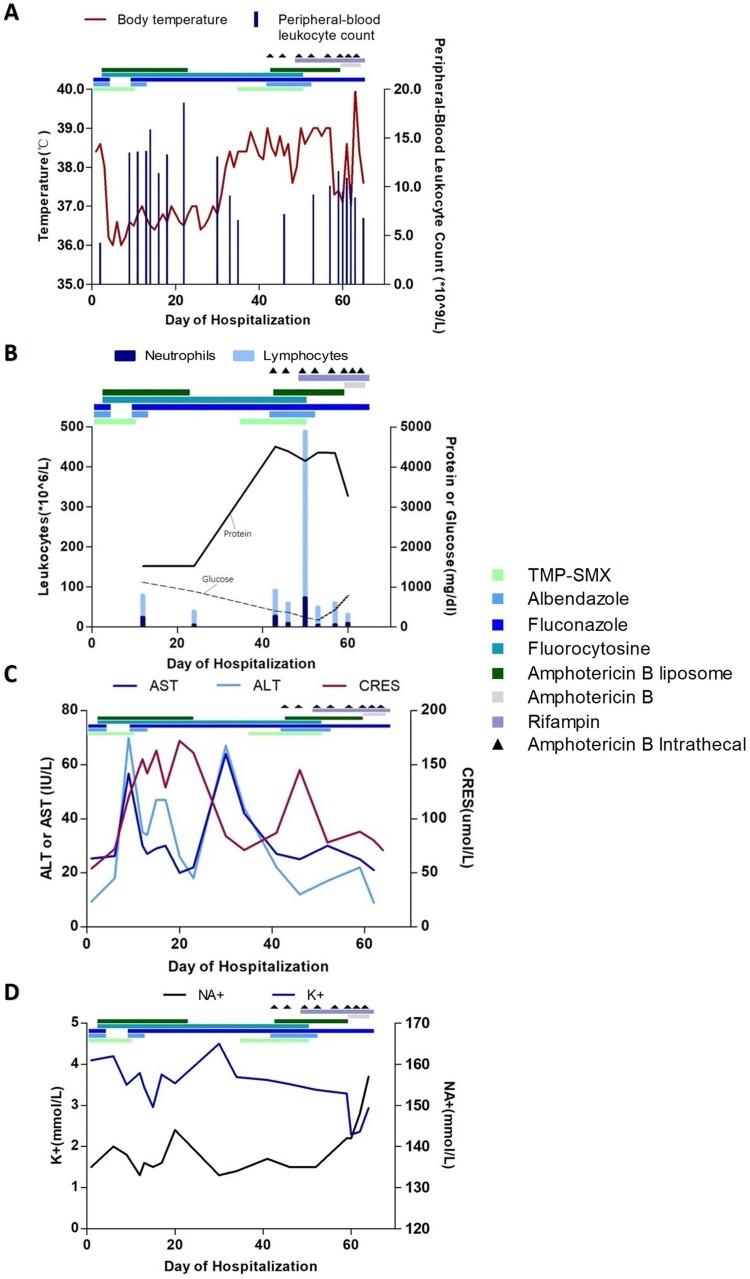
Clinical course of a 13-year-old girl with Balamuthia mandrillaris encephalitis. Graph A shows the body-temperature curve (red line) and peripheral-blood leukocyte counts (blue bars). Graph B shows the leukocyte count and differential (bars) and the protein (solid line) and glucose (dashed line) levels from cerebrospinal fluid samples. Graphs C and D show the liver and kidney function and serum electrolyte levels in serially collected blood samples. ALT: alanine aminotransferase; AST: aspartate aminotransferase; CR: creatinine.

Antibacterial therapy was discontinued, and the patient was placed on a regimen of albendazole (400 mg daily), trimethoprim-sulfamethoxazole (TMP-SMX; 72 mg/kg/day in three divided doses), and azithromycin (10 mg/kg/day). The patient was admitted in April with fever, a headache, vomiting and chest tightness. Upon admission, she was treated with albendazole (400 mg once daily) and fluconazole (400 mg once daily) for 3 days, but her situation did not improve. Her treatment was changed to amphotericin B liposome, 5-fluorocytosine and TMP-SMX (Table 1). Her symptoms subsequently improved, and her fever waned (Figure 5).

**Table 1.** Recommended drug treatment for Balamuthia mandrillaris encephalitis.

Drug	Dose	Route	Dutation
Amphotericin B liposome	0.1–0.5–1–1.5–2–2.5 mg/kg/day once daily.	IV	36 days
Amphotericin B	0.18–0.25–0.375–0.375–0.5 mg/kg/day once daily.	IV	5 days
Amphotericin B	0.025–0.05–0.075–0.1–0.2–0.4–0.5–0.6 mg every three days.	Intrathecal	8 days
TMP-SMX	30–36 mg/kg/day in two divided doses.	PO	24 days
Albendazole	400 mg once daily.	PO	16 days
Fluconazole	400 mg once daily.	IV	59 days
Fluorocytosine	100 mg/kg/day in four divided doses.	PO	48 days
Rifampin	10 mg/kg/day once daily.	PO	16 days

Note: Acronyms: IV-Intravenous; PO-Oral Therapy.

**Figure 6.**
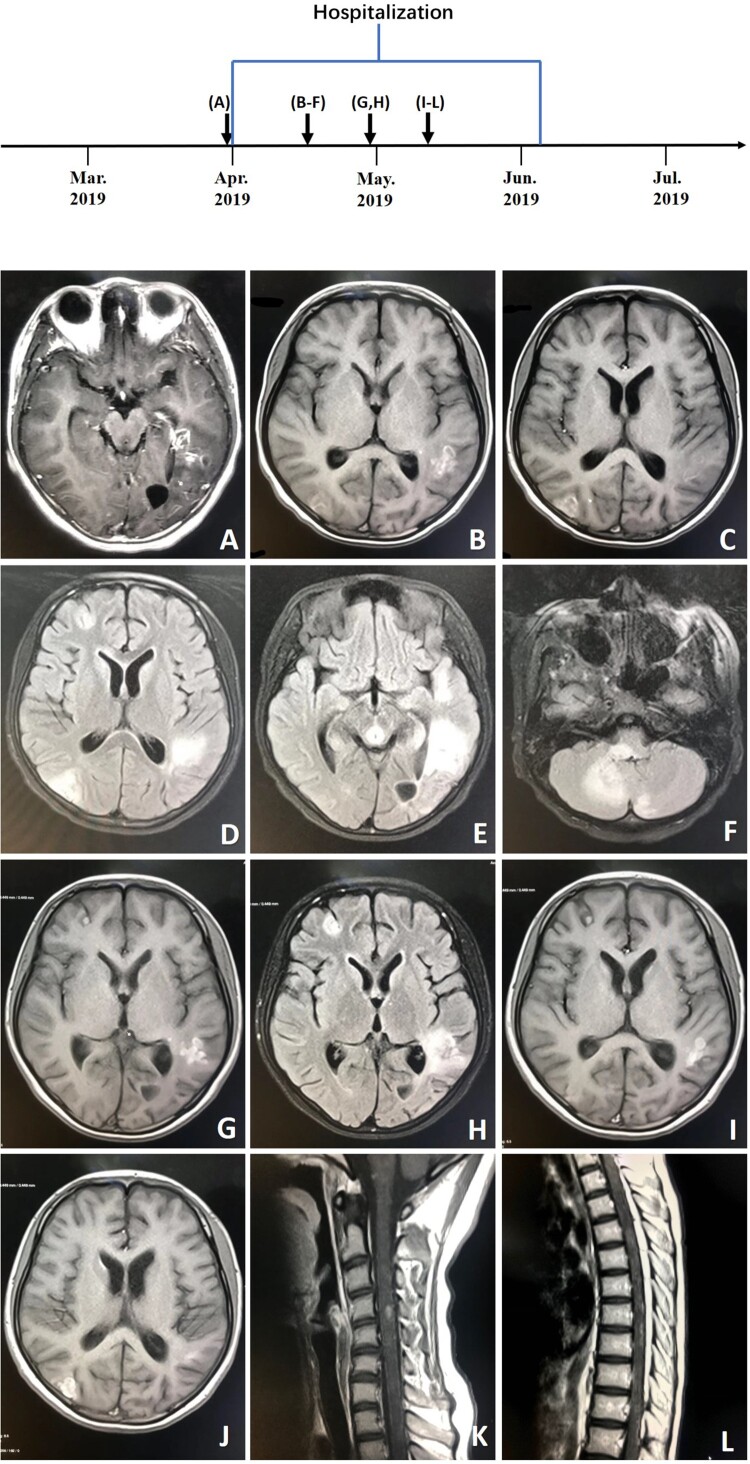
A, Axial T1-weighted enhanced image (March 28, 2019) showing multiple enhanced lesions throughout the brain, including the parietal lobe, left temporal lobe and occipital, and cerebellum. B, C, Axial T1-weighted images (April 13, 2019). D–F, Axial T2-weighted images (tirm dark fluid) (April 13, 2019) showing larger areas of multiple enhanced nodular lesions. G, Axial T1-weighted image (April 26, 2019). H, Axial T2-weighted image (tirm dark fluid) (April 26, 2019) showing new patchy lesions in the right frontal region. I–L, T1-weighted images (May 8, 2019) of the spinal cord and head showing new patchy lesions in the cervical and thoracic regions.

On day 9 of her hospitalization, the patient developed chest tightness and ventricular fibrillation, due to severe cardiovascular adverse reactions of amphotericin B liposome, and was placed on a ventilator. She was transferred directly to the intensive care unit and administered therapy consisting of amphotericin B liposome, 5-fluorocytosine and fluconazole. She improved and was taken off the ventilator 4 days later. On day 22 of her hospitalization, treatment with amphotericin B liposome was discontinued as her renal function progressively worsened. She was transferred to the department of infectious diseases in stable condition on day 24. At this time, her symptoms worsened, and she developed progressive loss of muscle strength in the lower extremities. Magnetic resonance imaging scans of the spinal cord and head showed new lesions (Figure 6). Intravenous amphotericin B liposome and intrathecal amphotericin were added to the regimen. The patient was transferred to the intensive care unit because her neurological status had deteriorated. She soon became unable to breathe and was placed on a ventilator. Unfortunately, her symptoms did not improve, and she went into a deep coma with a Glasgow Coma Scale score of 3. On day 64, the patient went into cardiac arrest and was given cardiopulmonary resuscitation. The next day, the patient was returned to Quan-Zhou Hospital at the request of her parents. Two weeks later, given the poor prognosis, her family decided to cease medical treatment, and she was pronounced dead.

The patient had no predisposing immunosuppression. Her whole-genome sequencing results were normal, and she denied any history of drinking river water or consuming raw meat.

## Discussion

Free-living amoebas are widely disseminated in the environment worldwide. Four genera of amoebas infect the central nervous system (CNS) in humans: *Naegleria*, *Acanthamoeba*, *Balamuthia*, and *Sappinia* [1]. Two CNS clinical syndromes are associated with free-living amoebas: primary amoebic meningoencephalitis (PAM) and GAE. PAM, caused by *N. fowleri*, is an acute haemorrhagic meningoencephalitis. GAE, caused by *Acanthamoeba spp.*, *B. mandrillaris*, and *Sappinia pedata*, is a rare subacute-chronic infection of the CNS.

The first infection of *B. mandrillaris* was reported in a pregnant mandrill baboon in the San Diego Wild Animal Park in 1989 [2]. Cases of human infections have been reported since 1990. To date, 30 cases of BAE have been identified in children worldwide (Table 2). BAE is almost always fatal because of delayed diagnosis and a lack of effective therapy. *B. mandrillaris* can be isolated from water and soil worldwide. Its life cycle comprises two stages: trophozoites and cysts. Trophozoites are the infective form and can infect humans via the skin or lungs and spread to the brain and other organs via haematogenesis [24,25]. Increasing numbers of organ transplant-transmitted *B. mandrillaris* show that it can be transmitted through organ transplantation [26,27]. *B. mandrillaris* most likely accesses the CNS through the blood-brain barrier (BBB). Jayasekera et al. showed that *B. mandrillaris* activates human brain microvascular endothelial cells to release high levels of interleukin (IL)-6 cytokines, and this host inflammatory response may play an important role in traversing the BBB [28]. *B. mandrillaris* causes infections primarily in immunocompromised persons but also in immunocompetent hosts, especially children [29]. Risk factors include a young age, Hispanic ethnicity, and exposure to contaminated soil or water [30]. The girl in our report was not immunosuppressed and she had history of playing barefoot on the beach during the summer.

**Table 2.** Demographic characteristics, clinical data, and therapeutic regimens for children of Balamuthia mandrillaris Infection.

Patient	Age, years	Sex	Region	Presenting clinical symptoms	Mode of diagnosis	Outcome	Reference
1	14	M	Venezuela	CNS:seizures and weakness on right arm and leg	Autopsy	died	[3]
2	1.5	M	California	CNS:Ataxia,cranial nervepolsy,vestibular cerebellar nystagmus	Serology,indirect immunofluorescence,PCR(brain)	died	[4]
3	3	F	California	CNS:Seizures,emesis	indirect immunofluorescence,PCR (brain,CSF)	died	[5]
4	7	M	California	CNS:Headache,focal seizures,cranial nerve palsy	Serology(brain)	died	[4]
5	7	M	California	CNS:Headache,neck stiffness,seizures, lethargy,cranial nerve palsy(XIth)	indirect immunofluorescence, PCR(CSF)	died	[4]
6	12	M	California	CNS:Headache,emesis,altered mental status	indirect immunofluorescence, PCR(brain)	died	[4]
7	schoolage	F	Spain	skin:cutaneous lesions on the face; CNS:progressive neurological involvement	brain necropsy	died	[6]
8	3	M	czech republic	CNS:tiredness,seizures,unconscious	brain biopsy	died	[7]
9	3	F	California	CNS:fatigue,emesis,tonic-clonic seizures,coma	Indirect immunofluorescencetest;brain necropsy	died	[8]
10	2	F	California	CNS:headache,lethargic,right-sided hemiparesis	brain necropsy	died	[6]
11	7	M	California	CNS:focal seizures,wide based gait,right cranial nerve, change in personality, lethargic	brain biopsy	died	[6]
12	2.5	M	texas	CNS:emesis,ataxia,seizures	CSF and serum for Balamuthia antibody titer	died	[6]
13	11	M	texas	CNS:vomiting,progressive lethargy,clumsiness,right hemiparesis,focal seizure	brain biopsy	died	[9]
14	0.6	F	Australia	CNS:seizures,left-sided weakness,	brain necropsy,immunofluorescent	died	[10]
15	6	F	Vermont	CNS:headache,stiff neck, nausea and vomiting	brain biopsy,immunofluorescent	died	[11]
16	5	M	Australia	Skin:mid facial granulomatous lesion; CNS:right sixth-nerve palsy,ataxia, nystagmus.	brain biopsy	died	[12]
17	8	M	Portugal	CNS:headache,vomiting,lethargy,	brain biopsy, PCR	died	[13]
18	2	F	Arizona	CNS:intermittent disequilibrium and worsening gait	brain biopsy	died	[14]
19	7	M	Peruvian	skin:asymptomatic papule on the dorsum of the nose; CNS:neurologic changes	skin biopsy	died	[15]
20	12	M	Argentina.	CNS:lethargy,right body hemiparesis	brain biopsy, Indirect immunofluorescencetest	died	[16]
21	5	M	Argentina.	Skin:nonspecific granulomatous lesions; CNS:clonic upper limb seizures, loss of consciousness	brain biopsy (histopathologic examination, Immunofluo)	died	[14]
22	3	F	Argentina.	CNS:clonic partial seizures,profound deterio ration in level of consciousness	brain biopsy (immunofluorescence)	died	[14]
23	6	M	Argentina.	skin:cutaneous lesions in the face; CNS:right body hemiparesis and consciousness deterioration	brain biopsy (Neuropathologic examination, immunofluorescence)	died	[14]
24	6	M	Pennsylvania	CNS:lethargy, hallucinations, ataxia, diplopia, nystagmus, and cranial nerve palsy	mNGS (CSF)	died	[17]
25	5	F	California	CNS:generalized seizures	brain biopsy (immunofluorescence)	survived	[18]
26	4	F	Georgia	CNS:headache,intermittent gait abnormality,lethargy,occasional seizures	brain biopsy (immunofluorescence)	died	[19]
27	13	F	Arizona	CNS:headache,left hemiparesis,Coma	brain biopsy (Histopathology)	died	[20]
28	4	M	Mississippi	CNS:Personality changes, loss of appetite, muscle twitching, headache, seizure	Autopsy	died	[21]
29	4	F	Thai	CNS:headache,ataxia	PCR (csf)	died	[22]
30	2	F	Arizona	CNS:ataxia,irritability,lethargy	Autopsy	died	[23]

BAE has a subacute-to-chronic course, and fewer than 10% of patients survive. BAE is difficult to diagnose because it lacks characteristic clinical symptoms, laboratory and neuroimaging findings. *B. mandrillaris* primarily affects two organ systems in humans: the skin and the CNS. The most common clinical features include fever, headache, vomiting, altered mental status, weakness, and seizures [31]. In some cases, cutaneous lesions precede BAE [12]. The United States case series reported that only 5% of patients had a cutaneous form of the disease [31], whereas majority of patients presented with a cutaneous lesion initially in the Peruvian case series [32]. Skin lesions may lead to an earlier diagnosis and treatment and may prevent progression to BAE. Unfortunately, many cases of BAE are diagnosed postmortem via autopsy or immunostained sections of the skin or brain tissue [31].

CSF specimens from patients with BAE have mildly elevated white blood cell counts with lymphocytes predominating, slightly low or normal glucose, and elevated or normal protein. Infected CSF resembles that of viral encephalitis or aseptic meningitis. CSF specimens from patients with BAE have significantly elevated levels of IL-6 and IL-8 [33]. In the CSF of patients with BAE, *Balamuthia* is rarely detected microscopically or by PCR; most patients are diagnosed via brain biopsy [31]. Neuroimaging findings can be uncharacteristic of BAE, including oedema, enhanced lesions, and multifocal lesions. The lesions can be located throughout the brain in no particular region and vary in size. The differential diagnoses for BAE include neurotuberculosis, neurocysticercosis, fungal infections, neoplasms, viral meningoencephalitis, and acute disseminated encephalomyelitis [5].

*B. mandrillaris* treatment is rarely studied. Owing to successful use in surviving patients, pentamidine, sulfadiazine, fluconazole, flucytosine, clarithromycin, azithromycin, and miltefosine (now available commercially in the US) antimicrobials (alone or in combination) are recommended for treating Balamuthia infections. Miltefosine is an antileishmanial and antineoplastic agent, which has been effective in treating patients with BAE. A recent study demonstrated that nitroxoline may be an important new treatment for *B.mandrillaris* [34]. Because of the difficulty in diagnosing BAE and the resultant delay in initiating antimicrobial therapy, majority of patients died in a short period after diagnosis of BAE. We review the previously reported cases of infections due to *Balamuthia mandrillaris* in children (Table 2). So far, only 1 child with BAE are reported to have survived. This patient presented with fever, generalized seizures, and MRI findings of edema in the left temporal area of the brain. A CT-guided brain biopsy was performed 19 days after initial presentation. examination revealed acute suppurative and necrotizing inflammation, and structures consistent with amoebas, which were identified as *B. mandrillaris* by indirect immunofluorescence staining. The patient was treated with clarithromycin, fluconazole, flucytosine, and thioridazine. And the patient remained stable, and had no gross neurologic sequelae. The diagnosis and antimicrobial therapy were initiated early enough to halt the course of disease, leading to survival of this girl. In our case, the immunocompetent patient had a two-year history of cutaneous lesions on her left thigh. Despite various empiric therapies, the skin lesions progressed to neurologic symptoms. Because of the late diagnosis, treatment was ineffective, and the patient died soon after being conclusively diagnosed with BAE. Early diagnosis and treatment are essential to increase the chances of survival.

*B. mandrillaris* is difficult to culture. Skin biopsies should be performed, and common histopathologic characteristics include diffuse granulomatous reactions on the reticular dermis, surrounding infiltration rich in lymphocytes and plasma cells, and multinucleated giant cells. Brain biopsies typically demonstrate parenchymal necrosis and granulomas with foamy macrophages and multinucleated giant cells accompanied by lymphocytes. Definitive diagnosis requires visualizing a trophozoite or cyst [35]. In the Peruvian cases, amebic trophozoites may be seen in two-thirds of the skin biopsies [32]. But the amebic trophozoites were scarce in number, and were difficult to identify. Differentiation of trophozoites from histiocytes requires experience in recognizing the characteristic of these parasites. In the United States case series, 18% (2/11) of cases with Balamuthia infections showed trophozoites in the meninges. None showed cysts in the meninges [35]. Because of low trophozoite and cyst densities, indirect immunofluorescence and PCR assays have been developed to rapidly identify BAE [36,37]. Nevertheless, in most countries, the lack of availability of indirect immunofluorescence and PCR tests for *B. mandrillaris* limits the opportunity for diagnosis. As shown in our case, BAE is difficult to diagnose in patients lacking typical symptoms, laboratory or neuroimaging findings. Our patient underwent a series of tests and was treated with various empiric therapies without improvement. She was finally correctly diagnosed via NGS of the CSF.

NGS may make it easier to diagnose difficult-to-diagnose infections, although the pan-pathogen detection technique has limitations in determining the sensitivity or detection limit for all organisms. NGS is especially suitable for difficult cases, and NGS of clinical samples can help diagnose complicated infectious diseases of atypical, rare, and novel aetiologies. Increasing microbes have been identified via NGS since its advent in 2004 [4,38–40], including S. erinaceieuropaei, Taenia solium, and nontuberculous mycobacterium. The samples for NGS analysis are generally flexible, including Cerebrospinal fluid, respiratory secretions, blood, pleural effusion, ascites, tissue, etc. The current cost of NGS is higher (3000 Ren Min Bi [RMB]) than culture test. The turnaround time is under 48 hours, significantly faster than that of pathogen cultures [41], and is less affected by prior antibiotic exposure.

Encephalitis causes significant mortality worldwide, and more than 50% of cases have no identified etiology [38]. NGS of the CSF may guide timelier diagnoses and more targeted treatments for encephalitis of unknown origin, which may improve clinical outcomes. NGS is an unbiased and rapid approach, which can detect all pathogens in a sample. In the UK cases, the diagnostic yield for NGS in encephalitis is 50%. Brown et al. [42] reviewed 44 undiagnosed cases of encephalitis in which NGS provided diagnosis. In 28 of the 44 cases, novel, rare or unexpected organisms were detected which are unlikely to have been detected using specific PCR assays. In a multicenter, prospective study [43], Wilson et al reported that among 58 infections of the nervous system, NGS identified 13 (22%) that were not identified by conventional testing, and NGS made concurrent diagnoses in 19. A total of 8 of these 13 diagnoses made solely by NGS had a clinical effect, with guiding treatment in 7 cases.

In the field of amoebic encephalitis, NGS are now available for diagnosis of *Acanthamoeba castellani*, *N. fowleri* [44], and *Balamuthia mandrillaris*. The reports concerning the use of NGS for diagnosis of amoebic encephalitis are comprised of single case reports. In 2008, Wang Q et al. [45] reported a case of PAM disease, which was successfully detected *N. fowleri* in the CSF specimen via NGS. *N. fowleri* infection was subsequently confirmed by PCR. In 2015, Greninger et al. [46] successfully detected *Balamuthia mandrillaris* in a Brain Biopsy specimen via NGS. Wilson et al. [47] identified *Balamuthia mandrillaris* in the CSF of a patient with undiagnosed encephalitis via NGS. Subsequently, PCR confirmed the presence of *B. mandrillaris* in CSF and brain tissue. Our patient was finally correctly diagnosed with Balamuthia mandrillaris encephalitis via NGS of the CSF. *B. mandrillaris* was confirmed by PCR of a skin sample and immunostaining of serum and CSF samples. Because of the late diagnosis, the skin lesions progressed to neurologic symptoms, and the patient died soon after being conclusively diagnosed with BAE. In fact, treatment was not effective in our patients, mainly because of the late diagnosis. NGS of CSF is likely to significantly improve the early diagnosis of neurologic infections, leading to better outcomes.

In summary, BAE confers a high mortality rate owing to its difficult diagnosis and delayed effective therapy. BAE should be considered in patients with subacute or chronic granulomatous meningoencephalitis, especially with skin lesions, when CSF shows mildly elevated leukocyte counts with a lymphocytic predominance, slightly low or normal glucose and elevated or normal protein, or when the patient responds poorly to antibiotic therapy. We present the first case of BAE in children diagnosed using NGS in China. NGS of the CSF may offer better outcomes, as it can lead to earlier diagnosis and intervention. This study highlighted the important clinical significance of NGS in providing more advantageous diagnostic testing for unexplained paediatric encephalitis, especially with rare or emerging pathogens.

## References

[1] Visvesvara GS, Moura H, Schuster FL. Pathogenic and opportunistic free-living amoebae: *Acanthamoeba* spp., *Balamuthia mandrillaris*, *Naegleria fowleri*, and *Sappinia diploidea*. FEMS Immunol Med Microbiol. 2007;50(1):1–26. 17428307 10.1111/j.1574-695X.2007.00232.x

[2] Visvesvara GS, Martinez AJ, Schuster FL, et al. Leptomyxid ameda, a new agent of amebic meningoencephalitis in humans and animals. J Clin Microbiol. 1990;28(12):2750–2756. 2280005 10.1128/jcm.28.12.2750-2756.1990 PMC268267

[3] Julio Martinez A, Elia Guerra A, Garcia-Tamayo J, et al. Granulomatous amebic encephalitis: a review and report of a spontaneous case from Venezuela. Acta Neuropathol. 1994;87(4):430–434. 8017178 10.1007/BF00313614

[4] Huang Z, Zhang C, Fang X, et al. Identification of musculoskeletal infection with non-tuberculous mycobacterium using metagenomic sequencing. J Infection. 2019;78(2):158–169. 10.1016/j.jinf.2018.10.002 30312646

[5] Glaser C, Schuster F, Yagi S, et al. Balamuthia amebic encephalitis-California, 1999–2007. Morb Mortal Wkly Rept. 2008;57(28):768–771. 18636064

[6] Cuevas PM, Smoje PG, Jofre ML, et al. Granulomatous amoebic meningoencephalitis by Balamuthia mandrillaris: case report and literature review. Rev Chilena Infectol. 2006;23(3):237–242. 16896497 10.4067/s0716-10182006000300007

[7] Kodet R, Nohynkova E, Tichy M, et al. Amebic encephalitis caused by Balamuthia mandrillaris in a Czech child: description of the first case from Europe. Pathol Res Pract. 1998;194(6):423–429. 9689651 10.1016/S0344-0338(98)80033-2

[8] Bakardjiev A, Azimi PH, Ashouri N, et al. Amebic encephalitis caused by *Balamuthia mandrillaris*: report of four cases. Pediatr Infect Dis J. 2003;22(5):447–452. 12792389 10.1097/01.inf.0000066540.18671.f8

[9] Roy SL, Atkins JT, Gennuso R, et al. Assessment of blood-brain barrier penetration of miltefosine used to treat a fatal case of granulomatous amebic encephalitis possibly caused by an unusual *Balamuthia mandrillaris* strain. Parasitol Res. 2015;114(12):4431–4439. 26329128 10.1007/s00436-015-4684-8 PMC4676568

[10] Hill CP, Damodaran O, Walsh P, et al. Balamuthia amebic meningoencephalitis and mycotic aneurysms in an Infant. Pediatr Neurol. 2011;45(1):45–48. 21723460 10.1016/j.pediatrneurol.2011.05.003

[11] Li Q, Yang XH, Qian J. September 2004: A 6-year-old girl with headache and stiff neck. Brain Pathol. 2005;15(1):93–95. 15779245 10.1111/j.1750-3639.2005.tb00109.x PMC8095996

[12] Reed RP, Cookeyarborough CM, Jaquiery AL, et al. Fatal granulomatous amoebic encephalitis caused by *Balamuthia mandrillaris*. Med J Aust. 1997;167(2):82–84. 9251693 10.5694/j.1326-5377.1997.tb138785.x

[13] Tavares M, Correia Da Costa JM, Carpenter SS, et al. Diagnosis of first case of Balamuthia amoebic encephalitis in Portugal by immunofluorescence and PCR. J Clin Microbiol. 2006;44(7):2660–2663. 16825409 10.1128/JCM.00479-06 PMC1489463

[14] Combs FJ, Erly WK, Valentino CM, et al. Best Cases from the AFIP: *Balamuthia mandrillaris* amebic meningoencephalitis. Radiographics. 2011;31(1):31–35. 21257931 10.1148/rg.311105067

[15] Valverde J, Arrese JE, Pierard GE. Granulomatous cutaneous centrofacial and meningocerebral amebiasis. Am J Clin Dermatol. 2006;7(4):267–269. 16901188 10.2165/00128071-200607040-00009

[16] Galarza M, Cuccia V, Sosa FP, et al. Pediatric granulomatous cerebral amebiasis: a delayed diagnosis. Pediatr Neurol. 2002;26(2):153–156. 11897483 10.1016/s0887-8994(01)00360-5

[17] Haston JC, Rostad CA, Jerris RC, et al. Prospective cohort study of next-generation sequencing as a diagnostic modality for unexplained encephalitis in children. J Pediatric Infect Dis Soc. 2019. 10.1093/jpids/piz032. PMC7457329 31107955

[18] Deetz TR, Sawyer MH, Billman G, et al. Successful treatment of Balamuthia amoebic encephalitis: presentation of 2 cases. Clin Infect Dis. 2003;37(10):1304–1312. 14583863 10.1086/379020

[19] Moriarty P, Burke C, Mccrossin D, et al. Balamuthia mandrillaris encephalitis: survival of a child with severe meningoencephalitis and review of the literature. J Pediatric Infect Dis Soc. 2014;3(1):e4–e9. 26624913 10.1093/jpids/pit033

[20] Shehab KW, Aboul-Nasr K, Elliott SP. Balamuthia mandrillaris granulomatous amebic encephalitis with renal dissemination in a previously healthy child: case report and review of the pediatric literature. J Pediatric Infect Dis Soc. 2018;7(3):e163–e168. 29096002 10.1093/jpids/pix089

[21] Centers for Disease Control and Prevention (CDC). Balamuthia mandrillaris transmitted through organ transplantation-Mississippi, 2009. Morb Mortal Wkly Rep. 2010;59(36):1165–1170. 20847719

[22] Krasaelap A, Prechawit S, Chansaenroj J, et al. Fatal Balamuthia amebic encephalitis in a healthy child: a case report with review of survival cases. Korean J Parasitol. 2013;51(3):335–341. 23864745 10.3347/kjp.2013.51.3.335 PMC3712108

[23] Stidd DA, Root B, Weinand ME, et al. Granulomatous amoebic encephalitis caused by *Balamuthia Mandrillaris* in an immunocompetent girl. World Neurosurg. 2012;78(715):e7–e12. 10.1016/j.wneu.2011.10.040 22120559

[24] Cope JR, Ratard RC, Hill VR, et al. The first association of a primary amebic meningoencephalitis death with culturable naegleria fowleri in tap water from a US treated public drinking water system. Clin Infect Dis. 2015;60(8):e36–e42. 25595746 10.1093/cid/civ017 PMC4627687

[25] Kemble SK, Lynfield R, Devries AS, et al. Fatal naegleria fowleri infection acquired in Minnesota: possible expanded range of a deadly thermophilic organism. Clin Infect Dis. 2012;54(6):805–809. 22238170 10.1093/cid/cir961

[26] Farnon EC, Kokko KE, Budge PJ, et al. Transmission of *Balamuthia mandrillaris* by organ transplantation. Clin Infect Dis. 2016;63(7):878–888. 27358357 10.1093/cid/ciw422

[27] Roy SL, Metzger R, Chen JG, et al. Risk for transmission of *Naegleria fowleri* from solid organ transplantation. Am J Transplant. 2014;14(1):163–171. 24279908 10.1111/ajt.12536 PMC4676565

[28] Jayasekera S, Matin A, Sissons J, et al. Balamuthia mandrillaris stimulates interleukin-6 release in primary human brain microvascular endothelial cells via a phosphatidylinositol 3-kinase-dependent pathway. Microbes Infect. 2005;7(13):1345–1351. 16027019 10.1016/j.micinf.2005.05.001

[29] Rowen JL, Doerr CA, Voegl H, et al. Balamuthia mandrillaris: a newly recognized agent for amebic meningoencephalitis. Pediatr Infect Dis J. 1995;14(8):705–710. 8532431

[30] Schuster FL, Glaser C, Honarmand S, et al. Balamuthia amebic encephalitis risk, hispanic Americans. Emerg Infect Dis. 2004;10(8):1510–1512. 15503402 10.3201/eid1008.040139 PMC3320424

[31] Cope JR, Landa J, Nethercut H, et al. The epidemiology and clinical features of Balamuthia mandrillaris disease in the United States, 1974–2016. Clin Infect Dis. 2019;68(11):1815–1822. 30239654 10.1093/cid/ciy813 PMC7453664

[32] Bravo FG, Seas C. Balamuthia Mandrillaris amoebic encephalitis: an emerging parasitic infection. Curr Infect Dis Rep. 2012;14(4):391–396. 22729402 10.1007/s11908-012-0266-4

[33] Schuster FL, Yagi S, Gavali S, et al. Under the radar: *Balamuthia* Amebic encephalitis. Clin Infect Dis. 2009;48(7):879–887. 19236272 10.1086/597260

[34] Laurie MT, White CV, Retallack H, et al. Functional assessment of 2,177 U.S. and international drugs identifies the quinoline nitroxoline as a potent amoebicidal agent against the pathogen *Balamuthia mandrillaris*. MBIO. 2018;9(5):e02051–18. 30377287 10.1128/mBio.02051-18 PMC6212833

[35] Guarner J, Bartlett J, Shieh W, et al. Histopathologic spectrum and immunohistochemical diagnosis of amebic meningoencephalitis. Mod Pathol. 2007;20(12):1230–1237. 17932496 10.1038/modpathol.3800973

[36] Schuster FL, Honarmand S, Visvesvara GS, et al. Detection of antibodies against free-living amoebae Balamuthia mandrillaris and Acanthamoeba species in a population of patients with encephalitis. Clin Infect Dis. 2006;42(9):1260–1265. 16586385 10.1086/503037

[37] Yagi S, Schuster FL, Visvesvara GS. Demonstration of Balamuthia and Acanthamoeba mitochondrial DNA in sectioned archival brain and other tissues by the polymerase chain reaction. Parasitol Res. 2007;102(2):211–217. 17899196 10.1007/s00436-007-0749-7

[38] Salzberg SL, Breitwieser FP, Kumar A, et al. Next-generation sequencing in neuropathologic diagnosis of infections of the nervous system. Neurol Neuroimmunol Neuroinflamm. 2016;3(4):e251. 27340685 10.1212/NXI.0000000000000251 PMC4907805

[39] Blauwkamp TA, Thair S, Rosen MJ, et al. Analytical and clinical validation of a microbial cell-free DNA sequencing test for infectious disease. Nat Microbiol. 2019;4(4):663–674. 30742071 10.1038/s41564-018-0349-6

[40] Fan S, Qiao X, Liu L, et al. Next-generation sequencing of cerebrospinal fluid for the diagnosis of neurocysticercosis. Front Neurol. 2018;9(471):1–7. 29971042 10.3389/fneur.2018.00471 PMC6018529

[41] Miao Q, Ma Y, Wang Q, et al. Microbiological diagnostic performance of metagenomic next-generation sequencing when applied to clinical practice. Clin Infect Dis. 2018;67(suppl 2):S231–S240. 30423048 10.1093/cid/ciy693

[42] Brown JR, Bharucha T, Breuer J. Encephalitis diagnosis using metagenomics: application of next generation sequencing for undiagnosed cases. J Infect. 2018;76(3):225–240. 29305150 10.1016/j.jinf.2017.12.014 PMC7112567

[43] Wilson MR, Sample HA, Zorn KC, et al. Clinical metagenomic sequencing for diagnosis of meningitis and encephalitis. N Engl J Med. 2019;380(24):2327–2340. 31189036 10.1056/NEJMoa1803396 PMC6764751

[44] Zysset Burri DC, Mueller N, Beuret C, et al. Genome-wide identification of pathogenicity factors of the free-living amoeba Naegleria fowleri. BMC Genomics. 2014;15(1):496–510. 24950717 10.1186/1471-2164-15-496 PMC4082629

[45] Wang Q, Li JM, Ji JK, et al. A case of Naegleria fowleri related primary amoebic meningoencephalitis in China diagnosed by next-generation sequencing. BMC Infect Dis. 2018;18(1):349–353. 30055569 10.1186/s12879-018-3261-z PMC6064090

[46] Greninger AL, Messacar K, Dunnebacke T, et al. Clinical metagenomic identification of Balamuthia mandrillaris encephalitis and assembly of the draft genome: the continuing case for reference genome sequencing. Genome Med. 2015;7:113–126. 26620704 10.1186/s13073-015-0235-2 PMC4665321

[47] Wilson MR, Shanbhag NM, Reid MJ, et al. Diagnosing *Balamuthia mandrillaris* Encephalitis with metagenomic deep sequencing. Ann Neurol. 2015;78(5):722–730. 26290222 10.1002/ana.24499 PMC4624031

